# NRF2-Related Epigenetic Modifications in Cardiac and Vascular Complications of Diabetes Mellitus

**DOI:** 10.3389/fendo.2021.598005

**Published:** 2021-06-25

**Authors:** Jie Wang, Mengjie Xiao, Jie Wang, Shudong Wang, Jingjing Zhang, Yuanfang Guo, Yufeng Tang, Junlian Gu

**Affiliations:** ^1^ School of Nursing, Cheeloo College of Medicine, Shandong University, Jinan, China; ^2^ Department of Cardiology, The First Hospital of Jilin University, Changchun, China; ^3^ Department of Cardiology, The First Hospital of China Medical University, and Department of Cardiology at the People’s Hospital of Liaoning Province, Shenyang, China; ^4^ Department of Orthopedic Surgery, The First Affiliated Hospital of Shandong First Medical University, Jinan, China

**Keywords:** NRF2, epigenetic modifications, NRF2 activators, diabetic cardiac complication, diabetic vascular complication

## Abstract

Diabetes mellitus (DM) is a highly prevalent chronic disease that is accompanied with serious complications, especially cardiac and vascular complications. Thus, there is an urgent need to identify new strategies to treat diabetic cardiac and vascular complications. Nuclear factor erythroid 2-related factor 2 (NRF2) has been verified as a crucial target for the prevention and treatment of diabetic complications. The function of NRF2 in the treatment of diabetic complications has been widely reported, but the role of NRF2-related epigenetic modifications remains unclear. The purpose of this review is to summarize the recent advances in targeting NRF2-related epigenetic modifications in the treatment of cardiac and vascular complications associated with DM. We also discuss agonists that could potentially regulate NRF2-associated epigenetic mechanisms. This review provides a better understanding of strategies to target NRF2 to protect against DM-related cardiac and vascular complications.

## Introduction

Diabetes mellitus (DM) is a metabolic disorder caused by genetic and environmental factors. DM is the third-largest non-communicable disease, following only cardiovascular diseases and malignant tumors (1, 2). The majority of DM-related morbidity and mortality is due to cardiac and vascular diseases triggered by long-term exposure to high blood glucose (3). These negative effects of hyperglycemia may persist even after achieving glycemic control, known as “metabolic memory”, which is related to epigenetic modifications (4, 5). Thus, it is an urgent requirement to find novel epigenetics-related treatment strategies to prevent and protect against DM-induced cardiac and vascular complications.

The pathological characteristics of DM and its related complications have been extensively investigated. Specifically, oxidative stress, apoptosis, and inflammation have been reported to be involved in the development of DM-induced complications. Notably, oxidative stress is regarded as a major factor in the pathogenesis of diabetic complications (6). Strong evidence indicates that epigenetics plays a significant role in regulating oxidative stress. Nuclear factor erythroid 2-related factor 2 (NRF2), encoded by *nfe2l2* (7), is one of the major regulators of oxidative stress (8). Numerous studies have explored the key role of NRF2-related epigenetic modifications in various disease models (9, 10). NRF2-correlated epigenetic modifications have been proposed to decrease the occurrence and progression of DM-related cardiac and vascular complications *via* inhibiting oxidative stress (11, 12). However, the exact effects of NRF2-related epigenetic modifications in DM and its related complications require further investigation. To date, there is limited literature focused on NRF2-related epigenetic modifications and NRF2 agonists in the treatment of DM-related cardiac and vascular complications (13–15). The purpose of this review is to provide a retrospective summary of NRF2-associated epigenetic modifications in DM-related cardiac and vascular complications and discuss NRF2 agonists that could potentially be used to regulate NRF2-associated epigenetic mechanisms.

## The Effects of NRF2-Related Epigenetic Modifications on the Regulation of Oxidative Stress

**Figure 1:**
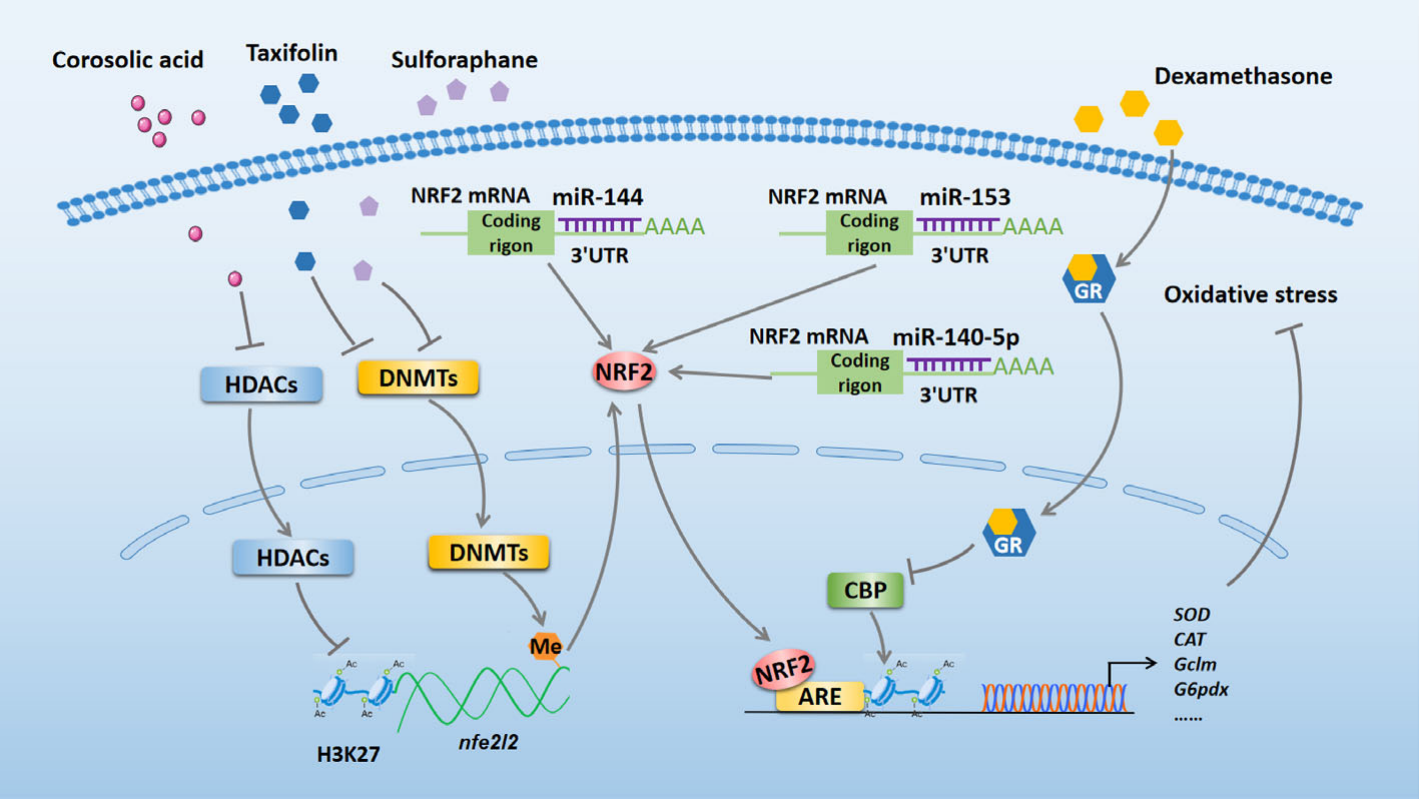
NRF2-related epigenetic mechanisms in the regulation of oxidative stress. Taxifolin and sulforaphane reduce DNA methylation of the *Nfe2l2* promoter region to exert antioxidant effect by inhibiting the expression of DNMTs. Corosolic acid increases acetylation of H3K27 in the *Nfe2l2* promoter region to exert antioxidant effect by inhibiting the expression of HDACs. Dexamethasone enhances GR recruitment to AREs to block NRF2-dependent CBP recruitment and histone acetylation at AREs, which inhibits the transcriptional activation of NRF2 target genes and reduces its antioxidant function. miR-140-5p, miR-153, and miR-144 bind to the 3’ UTR of NRF2 to aggravate oxidative stress by inhibiting NRF2 expression. NRF2, nuclear factor erythroid 2-related factor 2; miR, microRNA; DNMTs, DNA methyltransferases; HDACs, histone deacetylases; GR, glucocorticoid receptor; CBP, CREB-binding protein; ARE, antioxidant response element; SOD, superoxide dismutase; CAT, catalase; Gclm, glutamate-cysteine ligase modifier; G6pdx, glucose-6-phosphate dehydrogenase X-linked; 3’ UTR, 3’ untranslated region.

Many studies have demonstrated that oxidative stress is a major risk factor in multiple diseases (16, 17). Changes in epigenetic modifications can control phenotype and progression of diseases by modulating oxidative stress (18). Here, we review the literature regarding the effects of NRF2-related epigenetic modifications on the regulation of oxidative stress (summarized in **Figure 1**).

DNA methylation is a dynamically equilibrated process that is controlled by DNA methyltransferases (DNMTs) and DNA demethylation enzymes (19). A growing number of studies indicate that altered DNA methylation of *nfe2l2* plays an important role in regulating oxidative stress induced by various diseases. For example, in 12-O-tetradecanoylphorbol-13-acetate-induced carcinogenesis of mouse skin epidermal JB6P^+^ cells, reduced DNA methylation of the first 15 CpG sites in the *Nfe2l2* promoter region by taxifolin could suppress cellular oxidative stress *via* inhibiting the expression of DNMT1, DNMT3a, and DNMT3b (18). Consistent with this study, Zhao et al. reported that sulforaphane plays an antioxidative role by reducing DNA methylation of the *Nfe2l2* promoter through decreasing the levels of DNMTs in a cellular Alzheimer’s disease model (20). Conversely, in human ovarian cancer cells, increased DNA methylation on the *NFE2L2* promoter by the combined administration of trastuzumab and pertuzumab inhibited the expression of NRF2 and weakened its antioxidant function to perform an anti-cancer effect (21).

Apart from DNA methylation, histone modifications are key epigenetic mechanisms in the regulation of oxidative stress, as they affect gene expression by modifying the chromatin structure (22, 23). Recently, Yang et al. found that the administration of corosolic acid has an antioxidant effect in TRAMP-C1 prostate cells, which might be associated with increased acetylation of histone H3 lysine 27 (H3K27ac) and decreased trimethylation of H3K27 (H3K27me3) in the *Nfe2l2* promoter region. The authors proposed that corosolic acid exerts its effect by inhibiting protein expression of histone deacetylases (HDACs) (9). Another study using human embryonic kidney (HEK293T) and mouse hepatocellular carcinoma (Hepalclc7) cells pointed out that dexamethasone treatment enhanced glucocorticoid receptor (GR) recruitment to antioxidant response elements (AREs) region and subsequently blocked NRF2-dependent CREB-binding protein (CBP) recruitment and histone acetylation at AREs, which inhibited the transcriptional activation of NRF2 target genes and reduced its antioxidative function (23).

MicroRNAs (miRNAs) are 18–26 bp non-coding RNAs (ncRNAs) that have been reported to play a significant role in regulating oxidative stress (24, 25). Moreover, miRNAs have been verified to be involved in NRF2 regulation. Zhao et al. reported that miR-140-5p aggravated adriamycin-induced myocardial oxidative stress by inhibiting the expression of NRF2 (25). Similarly, miR-153 can increase reactive oxygen species production to further aggregate oxygen-glucose deprivation and reoxygenation-induced cardiomyocyte apoptosis by repressing the NRF2/heme oxygenase-1 (HO-1) signaling pathway (26). Furthermore, a reduction in NRF2 expression caused by miR-144 can exacerbate oxidative stress in erythrocytes from patients with homozygous sickle cell disease (27). Thus, therapeutic strategies targeting NRF2-associated epigenetic mechanisms may serve as effective approachs for treating various diseases related to oxidative stress.

## NRF2-Related Epigenetic Modifications in Diabetic Cardiac Complications

**Figure 2:**
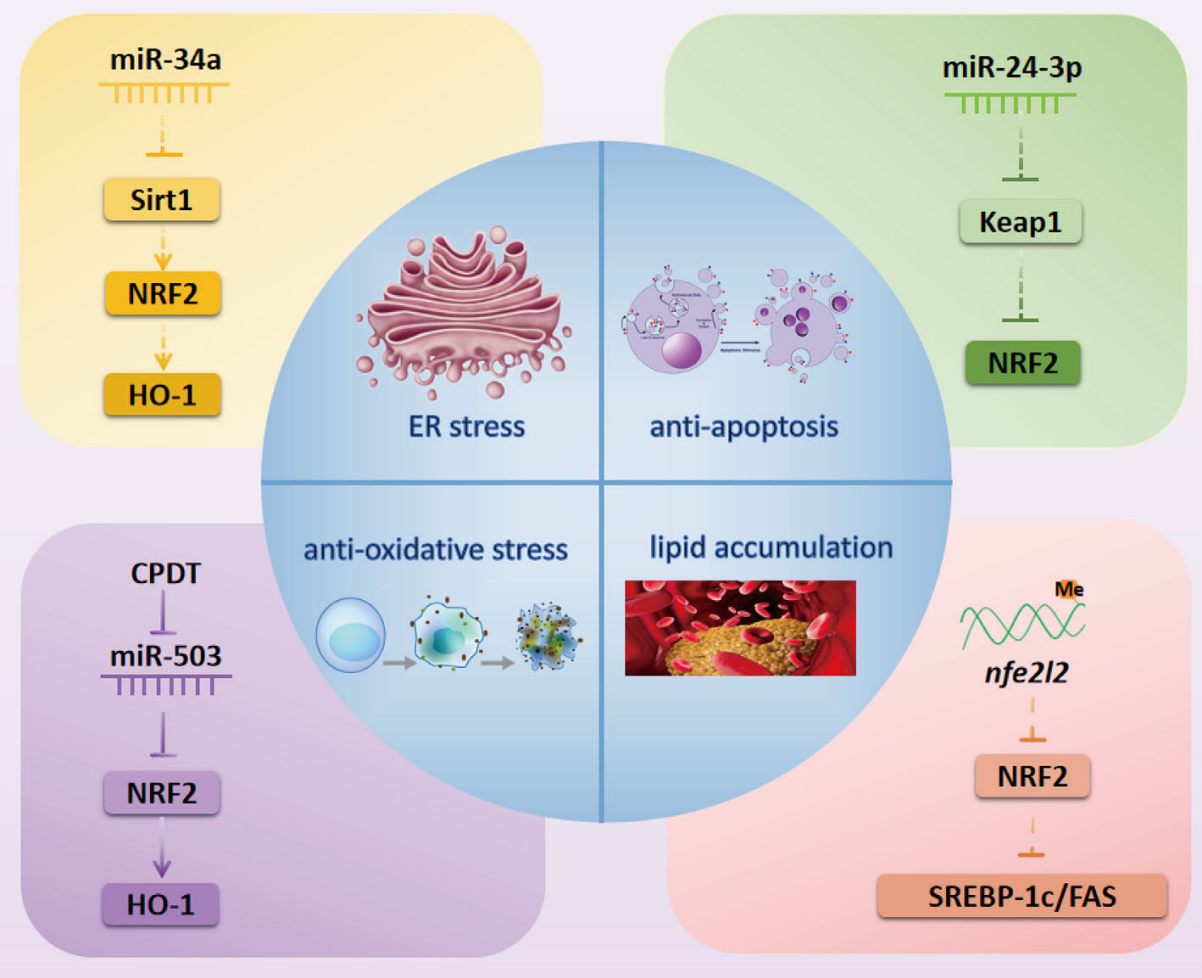
NRF2-related possibly epigenetic mechanisms in diabetic cardiac complications. Inactivation of the Sirt1/NRF2/HO-1 pathway by miR-34a might cause ER stress in diabetic myocardial I/R injury. miR-24-3p might activate NRF2 by inhibiting the expression of Keap1 to exert an anti-apoptosis effect in diabetic myocardial I/R injury. CPDT can activate the NRF2/HO-1 pathway by inhibiting miR-503 to reduce oxidative stress in DCM. Methylation of the *nfe212* promoter might inactivate NRF2 and its downstream targets SREBP-1c and FAS to cause lipid accumulation in DCM. NRF2, nuclear factor erythroid 2-related factor 2; miR, microRNA; Sirt1, Sirtuin1; HO-1, heme oxygenase-1; SREBP-1c, Sterol regulatory element-binding transcription factor 1c; FAS, fatty acid synthase; CPDT, 5,6-dihydrocyclopenta-1,2-dithiole-3-thione; Keap1, Kelch-like ECH-associated protein 1; ER, endoplasmic reticulum.

As one of the major complications of DM, diabetic cardiac complications are closely related to the occurrence of heart failure and poor prognosis of DM patients (28, 29). In recent years, a series of studies have confirmed that NRF2-related epigenetic modifications play a vital role in the prevention and treatment of diabetic cardiac complications (summarized in **Figure 2**).

### Diabetic Myocardial Ischemia-Reperfusion Injury

Sirtuin1 (Sirt1) is a nicotinamide adenine dinucleotide (NAD^+^)-dependent deacetylase that plays a vital role in regulating NRF2 expression in the treatment of diabetic myocardial ischemia-reperfusion (I/R) injury. For instance, Zhang et al. demonstrated that Sirt1 can enhance NRF2 nuclear translocation to prevent diabetic myocardial I/R injury in a diabetic Sprague Dawley rat model (30). Another study reported that Sirt1 deacetylated and reduced the ubiquitination of NRF2 in advanced glycation-end products-treated glomerular mesangial cells (GMCs) (31). These studies imply that deacetylation of NRF2 by Sirt1 might be a potential mechanism underlying NRF2 nuclear translocation. miRNAs have also been verified as common regulators of Sirt1. For example, crocin can relieve myocardial I/R-related endoplasmic reticulum (ER) stress through modulating the miR-34a/Sirt1/NRF2 signaling pathway in primary neonatal mouse cardiomyocytes (32). Thus, we further speculate that the miR-34a/Sirt1/NRF2 pathway might be involved in the prevention and treatment of diabetic myocardial I/R injury. Moreover, Xiao et al. further demonstrated that luteolin modulates the Kelch-like ECH-associated protein 1 (Keap1)/NRF2 axis to inhibit oxidative stress, thus attenuating diabetic myocardial I/R injury (33). miR-24-3p has also been shown to regulate the Keap1/NRF2 pathway in myocardial I/R injury (34). Thus, it is possible that miR-24-3p may act upstream of Keap1/NRF2 pathway to alleviate diabetic myocardial I/R injury.

### Diabetic Cardiomyopathy

NRF2-associated epigenetic modifications have been shown to act as crucial mechanisms in diabetic cardiomyopathy (DCM), with some literature indicating that epigenetic modifications can promote DCM, while other studies suggest that they inhibit DCM.

Methylation of the *nfe2l2* promoter might be a potential contributor to the incidence and development of DCM. Wang et al. reported that cardiac NRF2 expression was significantly decreased in a DM mouse model (35). Meanwhile, another study showed that the methylation level of the *Nfe2l2* promoter was increased but the gene and protein expression of NRF2 was decreased under high glucose (HG) condition (36). Thus, we speculate that decreased expression of NRF2 may be related to methylation of the *nfe2l2* promoter in DCM.

ncRNAs also play a major role in DCM through targeting NRF2. In HG-stimulated rat and mouse cardiomyocytes models, the inhibition of miR-144, miR-155, and miR-503 can active NRF2 to attenuate cellular oxidative stress and reduce cardiomyocyte apoptosis to prevent DCM (12, 37, 38). Recently, Gao et al. found that long ncRNA (lncRNA) HOX transcript antisense RNA (HOTAIR) and Sirt1 were downregulated but miR-34a was upregulated in diabetic hearts and HG-stimulated H9c2 cells, while overexpression of lncRNA HOTAIR protected against DCM *via* increasing Sirt1 expression by sponging miR-34a (39). Furthermore, the Sirt1/NRF2 pathway was shown to play a role in improving DCM *via* alleviating myocardial oxidative stress (40). However, whether NRF2 can be regulated by the lncRNA HOTAIR/miR-34a/Sirt1 pathway in the treatment of DCM needs to be further explored.

## NRF2-Related Epigenetic Modifications in Diabetic Vascular Complications

**Figure 3:**
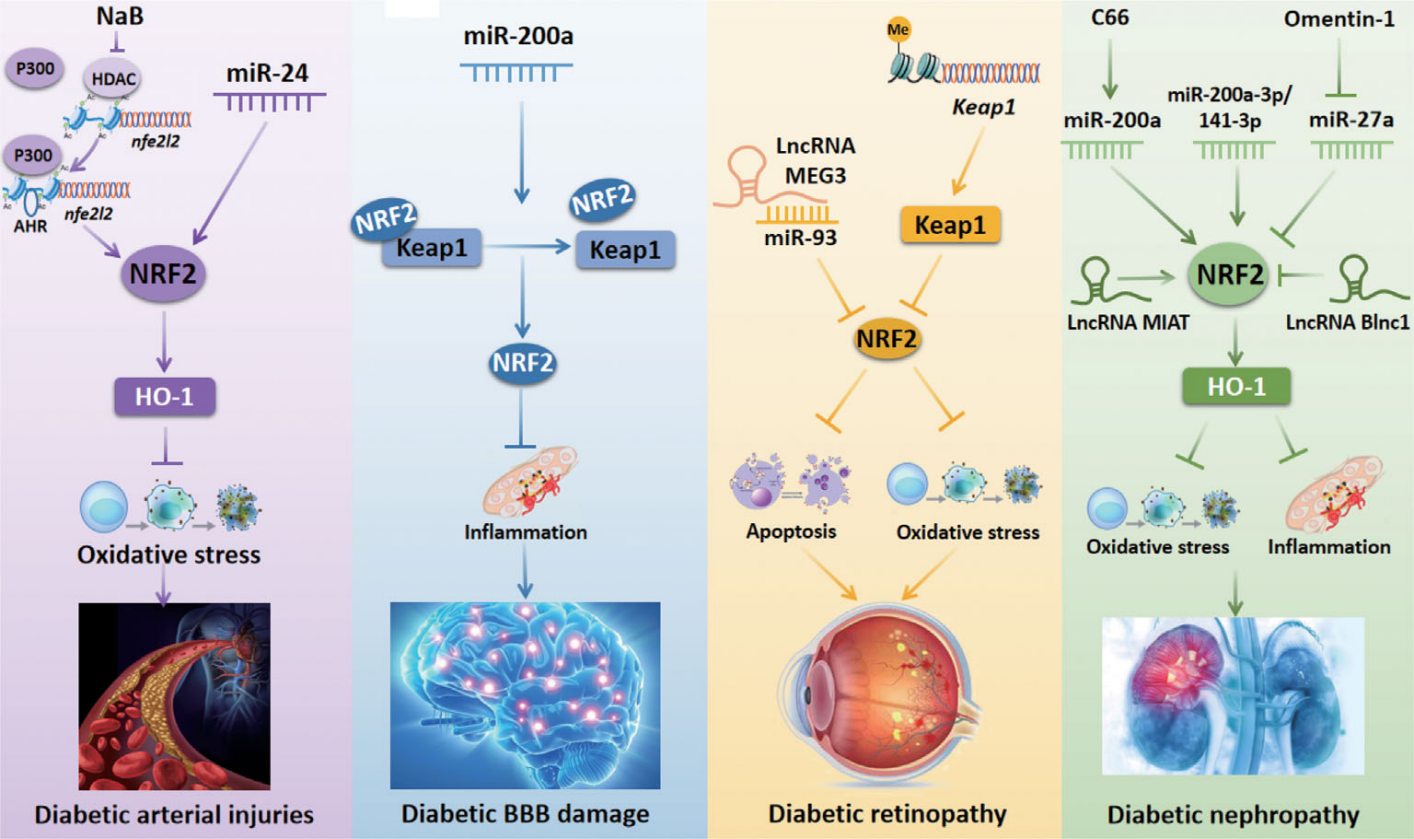
NRF2-related epigenetic mechanisms in diabetic vascular complications. Inhibition of HDAC activity by NaB increases the occupancy of AHR and P300 at *nfe2l2* promoter to promote the transcriptional activation of *nfe2l2*, thus protecting against diabetic arterial injuries. miR-24 stimulates the NRF2/HO-1 signaling pathway to prevent oxidative stress induced by diabetic arterial injuries. miR-200a regulates the Keap1/NRF2 axis to prevent inflammation, thus improving diabetic BBB damage. The lncRNA MEG3 inhibits DR-induced apoptosis *via* downregulating the miR-93/NRF2 pathway. Histone methylation of the Keap1 promoter region increases Keap1 expression and subsequently inhibits the activity of NRF2 to aggravate oxidative stress in DR. C66 upregulates NRF2 expression to protect against DN-induced oxidative stress by increasing miR-200a expression. The upregulation of miR-200a-3p/141-3p modulates the NRF2 to protect against DN. Omentin-1 downregulates miR-27a and subsequently increases NRF2 expression to inhibit oxidative stress and inflammation in DN. The lncRNA MIAT improves DN by stimulating NRF2. The lncRNA Blnc1 reduces NRF2 expression to cause oxidative stress and inflammation in DN. NaB, sodium butyrate; HDAC, histone deacetylase; AHR, aryl hydrocarbon receptor; NRF2, nuclear factor erythroid 2-related factor 2; miR, microRNA; lncRNA MIAT, long non-coding RNA myocardial infarction-associated transcript; HO-1, heme oxygenase-1; Keap1, Kelch-like ECH-associated protein 1; BBB, blood-brain barrier.

Diabetic vascular complications can be divided into macrovascular and microvascular complications. The macrovascular complications include cardiovascular diseases, cerebrovascular diseases, and peripheral vascular diseases. The microvascular complications include diabetic retinopathy (DR) and diabetic nephropathy (DN). NRF2-related epigenetic modifications have been verified to exert a key role in diabetic vascular complications (summarized in **Figure 3** **)**.

### DM-Related Arterial Injuries

Arterial injury is a common vascular complication in patients with DM. Endothelial dysfunction is the critical manifestation of diabetic vascular complications, followed by increased oxidative stress and inflammation (41). Sodium butyrate (NaB) is an HDAC inhibitor that has been shown to increase the occupancy of both *Nfe2l2*’s transcription factor aryl hydrocarbon receptor (AHR) and histone acetylase P300 at the promoter region of the *Nfe2l2*. Binding of these factors promotes the transcriptional activation of *Nfe2l2*, thus protecting against aortic endothelial dysfunction in HG-treated endothelial cells (ECs) (41). Some studies also revealed that NRF2 regulation by miRNAs may play a critical role in diabetic arterial injuries. For instance, miR-24 has been shown to stimulate the NRF2/HO-1 signaling pathway to prevent oxidative stress in HG-stimulated vascular smooth muscle cells (VSMCs) (42). In human umbilical vein ECs treated with oxidized low-density lipoprotein, miR-140-5p can suppress the expression of NRF2 to aggravate atherosclerosis (AS)-related oxidative stress (43). Additionally, Hur et al. have shown that the NRF2/ARE axis can activate the downstream antioxidant enzyme NADPH quinone oxidoreductase-1 (NQO-1) in VSMCs treated with HG and in a rat model of DM-induced AS (44). These studies provide evidence that upregulation of the NRF2/ARE axis by the inhibition of miR-140-5p may emerge as a potential therapeutic strategy for treating diabetic AS.

In both HG-treated GMCs and diabetic mice, upregulation of Sirt1 expression can activate NRF2 (45). Meanwhile, another study indicated that the inhibition of miR-217 could enhance Sirt1 expression in oxidized low−density lipoprotein treated THP-1 cells (46). Therefore, we speculate that miR-217 might modulate the Sirt1/NRF2 pathway in diabetic AS progression. Furthermore, a study by Xie et al. showed that Keap1 sulfhydrylation at Cys151 and NRF2 activation by hydrogen sulfide could inhibit oxidative stress to attenuate DM-induced AS both *in vitro* and *in vivo*, which indicates that Keap1/NRF2 signaling is a critical regulator of diabetic AS (47). The Keap1/NRF2 pathway has also been reported to be regulated by miR-200a to ameliorate oxidative stress in a type II DN rat model, which highlights the important function of miR-200a in the regulation of Keap1/NRF2 pathway (48). Based on the literature reviewed here, we suppose that the miR-200a/Keap1/NRF2 signaling pathway could be a new pharmacological target to prevent diabetic AS.

### Diabetic Blood-Brain Barrier Disruption

The blood-brain barrier (BBB) is the main internal barrier of the human body. It is responsible for regulating the neural microenvironment, as well as maintaining the stability of the intracerebral environment and the normal physiological state of the central nervous system (49, 50). Recently, a series of studies suggested that NRF2-related epigenetic modifications may exert antioxidant and anti-inflammatory effects to prevent diabetic BBB injury. Zhao et al. found that the inhibition of HDAC3 activated the miR-200a/Keap1/NRF2 signaling pathway to attenuate the inflammatory response, thus ameliorating diabetic-induced BBB permeability (51). Besides, the Keap1/NRF2 axis has been reported to be regulated by miR-200a-3p/141-3p in diabetic renal mesangial cells, suggesting that these miRNAs are involved in reducing oxidative stress and protecting against DN (52). However, whether miR-200a-3P/141-3P can regulate the Keap1/NRF2 axis to prevent diabetic BBB damage needs to be further explored.

### Diabetic Retinopathy

DR is one of the most common diabetic microvascular complications, which is a major cause of severe vision loss in individuals with DM due to retinal microangiopathy (53). ncRNAs have been reported to be associated with a risk of DR, and an increasing number of studies have focused on the effects of ncRNAs on NRF2 in the progression of DR. A recent study showed that expression of the lncRNA MEG3 and NRF2 was reduced, but the expression of miR-93 was elevated in blood samples from DR patients as well as in HG-treated human retinal pigment epithelium (RPE) and ARPE-19 cells. However, overexpression of lncRNA MEG3 inhibited apoptosis of RPE cells treated with HG *via* downregulating miR-93. This study also indicated that NRF2 is negatively regulated by miR-93 (54). In contrast, knockdown of the lncRNA Sox2OT plays an anti-oxidative role *via* upregulating NRF2/HO-1 signaling in retinal ganglion cells exposed to HG (55). Besides, histone modifications at the promoter regions of the retinal genes are important in regulating NRF2. Mishra et al. investigated the role of epigenetic modifications at *Keap1* promoter in regulating NRF2 function. They found that hyperglycemia increased the binding of stimulating protein-1 (Sp1) at the *Keap1* promoter, increased monomethylation of H3K4 (H3K4me1), and activated methyltransferase enzyme Set7/9. Further analysis showed that the inhibition of histone methylation of the *Keap1* promoter region decreased Keap1 expression and subsequently enhanced the activity of NRF2 to attenuate DR (56). They also investigated the role of epigenetic modifications in the decreased NRF2 binding at glutamate-cysteine ligase catalytic subunit (*Gclc*)-*ARE4* and showed that H3K4me2 at *Gclc-ARE4* was elevated, H3K4me3 and H3K4me1 as well as NRF2 binding at *Gclc-ARE4* were reduced in DM. Histone demethylase (LSD1) siRNA increased H3K4me1 at *Gclc-ARE4* and enhanced NRF2 binding at *Gclc-ARE4* and Gclc transcripts (57). However, the relationship between H3K4me1 at *Gclc-ARE4* and NRF2 binding at *Gclc-ARE4* needs to be further investigated. The above findings provide a novel perspective for the treatment of DR.

### Diabetic Nephropathy

DN is a complication of type I and type II DM caused by microvascular changes (58, 59). In recent years, accumulating studies have confirmed that NRF2 prevents DN progression *via* epigenetic modifications. Inhibition of HDAC by NaB has been proposed to activate *Nfe2l2* gene transcription to ameliorate DN by enhancing transcription factor binding to the promoter region of the *Nfe2l2* gene (60). Song et al. demonstrated that omentin-1 can decrease the expression of inflammatory markers interlukin-8, monocyte chemotactic protein 1, and tumor necrosis factor-α, and the oxidative stress marker malondialdehyde, as well as elevate antioxidant enzymes catalase and superoxide dismutase (SOD). Further mechanistic analysis revealed that omentin-1 can downregulate miR-27a to increase the expression of NRF2 by reducing miR-27a binding at the NRF2 3’ untranslated region (UTR) in type II DN (61).

Another study by Wu et al. indicated that miR-200a is a pivotal factor that mediates the expression of NRF2 in the treatment of DN. They reported that renal expression of NRF2 and miR-200a was downregulated, but the expression of Keap1 was upregulated in diabetic mice. However, C66 downregulated Keap1 expression and subsequently upregulated NRF2 expression to protect against DN-induced albuminuria, oxidative damage, and fibrosis by increasing miR-200a expression (62). Opposite with the above finding, Civantos et al. found that the miR-200a/Keap1/NRF2 pathway was diminished following treatment with sitagliptin to ameliorate oxidative stress induced by DN (48). However, more studies regarding these controversial findings are needed to achieve a more comprehensive understanding. Moreover, aldose reductase deficiency was shown to upregulate miR-200a-3p/141-3p to regulate Keap1/NRF2 signaling pathway to protect against DN (52). LncRNAs have also been found to regulate NRF2 in DN. The lncRNA myocardial infarction-associated transcript (MIAT) has been shown to improve HG-induced renal tubular epithelial injury by stimulating NRF2 (63). To the contrary, the expression of NRF2 was decreased by the lncRNA Blnc1 in HK-2 cells exposed to HG (64). Thereby, the regulation of NRF2 by lncRNAs should be further explored as potential therapeutics to reduce the burden of DN.

## Epigenetic Modifications of NRF2 by Pharmacological Agents

Emerging studies have indicated that aberrant DNA methylation of *nfe2l2* serves as a crucial driving factor in the occurrence and development of various diseases (36). Many antioxidant compounds have been proposed to regulate *nfe2l2* expression by modulating DNA methylation at the CpG sites of the promoter sequence of *nfe2l2*. Resveratrol, widely found in grapes, mulberries, peanuts, and red wine, is a natural phenolic compound with strong anti-inflammatory, antioxidant, and anti-cancer properties (65–68). Resveratrol was shown to prevent HG-induced oxidative stress in HepG2 cells *via* suppressing methylation of *Nfe2l2*, which attenuated HG-induced triglyceride accumulation (36). Sulforaphane (SFN), found in cruciferous vegetables like broccoli, bok-choy, and cabbage, has also been reported to block methylation of the *Nfe2l2* promoter, thereby enhancing its transcriptional activity (20, 69–72). For instance, SFN can decrease the protein expression of DNMT1 and DNMT3a and induce demethylation of the first 5 CpGs in the *Nfe2l2* promoter region. This finding was corroborated by increased mRNA and protein expression of NRF2 and its downstream target gene NQO-1 in TRAMP-C1 cells, suggesting that SFN might protect against prostate cancer-induced oxidative stress (72). In addition, luteolin, a flavonoid compound that is isolated from bird’s eye chilli, onions, carrots, and olive oil (73), can enhance DNA demethylation of the *NFE2L2* promoter to protect against oxidative stress in human colorectal cancer HCT116 cells (74). Moreover, pelargonidin, fucoxanthin, tanshinone IIA, reserpine, and delphinidin have been used in the treatment of skin cancer, while curcumin, γ-tocopherol-rich mixture of tocopherols (γ-TmT) and 3,3’-diindolylmethane (DIM) have been proposed to prevent prostate cancer; all of these protective effects are mediated by decreasing methylation of the *Nfe2l2* promoter (75–82). Therefore, therapeutic strategy targeting the demethylation of the *nfe2l2* promoter region may be an effective method to attenuate oxidative stress.

As a potential treatment strategy for many diseases, histone modifications-based therapies have gained significant interest. The protection conferred by HDAC inhibition may be associated with the upregulation of histone acetylation at the promoter region of *nfe2l2*. NaB, a natural short-chain fatty acid, is an HDAC inhibitor that affects proliferation, differentiation, and apoptosis of cell (83). Dong et al. demonstrated that NaB may activate *Nfe2l2* at the transcriptional level to ameliorate DN possibly by inhibiting HDAC activity (60). They subsequently found that NaB can decrease oxidative stress and inflammatory response in the aorta. Specifically, NaB suppressed the activity of HDAC and increased the interaction of AHR and P300 at the *Nfe2l2* promoter to increase the expression of NRF2, alleviating DM-related aortic endothelial dysfunction (41). Additionally, corosolic acid, a triterpenoid found in various plants, such as *Schisandra chinensis, Lagerstroemia speciosa L., and Weigela subsessilis* (84), is reported to have anti-cancer activity. One study investigated the effects of corosolic acid on NRF2 *via* epigenetic modifications and found that it exerts its antioxidant effect by increasing H3K27ac and decreasing H3K27me3 at the *Nfe2l2* promoter region in TRAMP-C1 prostate cells (9). Another study investigated the effect of the epigenetic regulator Set7/9 in modulating NRF2 expression and found that Set7/9 knockdown reduced H3K4me1 enrichment at the promoter region of *NFE2L2*, while treatment with two phytochemicals, phenethyl isothiocyanate (PEITC) and ursolic acid (UA), elevated the enrichment (85). Collectively, the above findings emphasize the importance of NRF2 agonists and their epigenetic effects on the *Nfe212* promotor in the prevention of multiple diseases.

NRF2 agonists could confer their protection *via* targeting miRNAs. For example, NRF2 agonists could reduce the direct effect of miRNAs on NRF2. Song et al. revealed that omentin-1, a novel adipocytokine (86), reduced oxidative stress and inflammatory response to improve the deterioration of DN *via* downregulating the expression of miR-27a, reducing the binding of miR-27a at the 3’ UTR of NRF2, and significantly increasing NRF2 expression (61). As a complex enzyme, the phase II enzyme inducer (CPDT) promotes the expression of HO-1 through activating NRF2 to ameliorate DCM. Specifically, CPDT treatment was shown to decrease miR-503 expression and then upregulate the expression of NRF2 (12). However, miRNAs can also indirectly regulate NRF2. For example, C66 and zopolrestat act on the 3’UTR of Keap1, further impeding transcriptional activity of *Nfe2l2* (52, 62). Thus, treatment strategy targeting miRNA-related epigenetic modifications of NRF2 could potentially prevent the progression of various DM-related diseases. Pharmacological agents reported to regulate NRF2 signaling epigenetically have been summarized in **Table 1**.

**Table 1:** Pharmacological agents reported to regulate NRF2 signaling epigenetically.

Reference	Pharmacological agents	Cell/Animal type	Epigenetic mechanism
(36)	Resveratrol	HepG2 cells and C57/BL6 mice	decrease methylation of *Nfe2l2*
(72)	SFN	TRAMP-C1 cells	decrease methylation of *Nfe2l2*
(76)	Curcumin	TRAMP-C1 cells	decrease methylation of *Nfe2l2*
(81)	γ-TmT	TRAMP-C1 cells and TRAMP mice	decrease methylation of *Nfe2l2*
(82)	DIM	TRAMP-C1 cells and TRAMP mice	decrease methylation of *Nfe2l2*
(74)	Luteolin	HCT116 cells	decrease methylation of *Nfe2l2*
(75)	Pelargonidin	JB6 P^+^ cells	decrease methylation of *Nfe2l2*
(77)	Fucoxanthin	JB6 P^+^ cells	decrease methylation of *Nfe2l2*
(78)	Tanshinone IIA	JB6 P^+^ cells	decrease methylation of *Nfe2l2*
(79)	Reserpine	JB6 P^+^ cells	decrease methylation of *Nfe2l2*
(80)	Delphinidin	JB6 P^+^ cells	decrease methylation of *Nfe2l2*
(41)	NaB	ECs and C57BL/6 mice	inhibit HDAC activity
(60)	NaB	C57BL/6 mice	inhibit HDAC activity
(9)	Corosolic acid	TRAMP-C1 cells	increase H3K27ac and decrease H3K27me3 at the promoter region of *Nfe2l2*
(85)	PEITC and UA	PCa LNCaP and PC3 cell lines	increase H3K4me1 enrichment at the promoter region of *NFE2L2*
(61)	Omentin-1	NRK-52E, HK-2, HBZY-1 cell lines and C57BLKS/JNju mice	decrease miR-101 targeting 3’ UTR of NRF2
(12)	CPDT	Primary myocardial cells and Wistar rats	decrease miR-503 targeting 3’ UTR of NRF2
(62)	C66	C57BL/6 mice	increase miR-200a targeting 3’UTR of *Keap1* to activate NRF2 signaling
(52)	Zopolrestat	Mouse mesangial SV40-Mes13 cells and C57BL/6 mice	increase miR-200a-3p/141-3p targeting 3’UTR of *Keap1* to activate NRF2 signaling

SFN, sulforaphane;γ-TmT, γ-tocopherol-rich mixture of tocopherols;

DIM, 3,3’-diindolylmethane; NaB, sodium butyrate; PEITC, phenethyl isothiocyanate; UA, ursolic acid; CPDT, phase II enzyme inducer; NRF2, nuclear factor erythroid 2-related factor 2; HDAC, histone deacetylase; H3K27ac, acetylation of histone H3 lysine 27; H3K27me3, trimethylation of H3K27; H3K4me1, monomethyl H3K4; 3’ UTR, 3’ untranslated region; Keap1, Kelch-like ECH-associated protein 1; ECs, endothelial cells; miR, microRNA.

## Conclusion

Epigenetic regulation plays a crucial role in DM-related cardiac and vascular complications. NRF2-related epigenetic modifications have evolved as a novel research direction for the treatment of multiple diseases. Therefore, this review highlights the effects of NRF2-associated epigenetic mechanisms (DNA methylation, histone methylation and acetylation, and regulation of miRNAs and lncRNAs) on DM-induced cardiac and vascular complications. However, the literatures focused on DNA methylation of *nfe2l2* in the treatment of DM-related cardiac and vascular diseases are limited, and the mechanisms by which miRNAs exert their direct effects on NRF2 have been largely lacking. Current literatures indicate that NRF2 agonists have anti-cancer effects, but more studies are needed to understand the role of these agonists in treating DM-induced cardiac and vascular diseases. In conclusion, this review highlights the importance of the NRF2-related epigenetic regulation in diabetic cardiac and vascular complications.

## Author Contributions

Study concept and design: All authors. Drafting of the manuscript: JW (ist author), MX, and JW (3rd author). Critical revision of the manuscript for important intellectual content: JG, YG, and YT. Obtained the funding: JG, JZ, and SW. All authors contributed to the article and approved the submitted version.

## Funding

This study was supported by Qilu Young Scholar’s Program of Shandong University (21330089963007), National Natural Science Foundation of China (81700329, 81770375), and Jilin Science and Technology Department (20200801061GH).

## Conflict of Interest

The authors declare that the research was conducted in the absence of any commercial or financial relationships that could be construed as a potential conflict of interest.

The reviewers ZJ & SZ declared a shared affiliation with one of the authors, SW, to the handling editor at time of review.
